# Characterizing financial risk from out‐of‐pocket expenditures across dementia stages

**DOI:** 10.1002/alz.70666

**Published:** 2025-09-15

**Authors:** Patricia G. Synnott, Yingying Zhu, Angie Mae Rodday, Pei‐Jung Lin

**Affiliations:** ^1^ Tufts Medical Center Boston Massachusetts USA

**Keywords:** catastrophic health expenditures, dementia, financial risk, health & retirement study, impoverishment, out‐of‐pocket costs

## Abstract

**INTRODUCTION:**

Older adults with dementia incur considerable out‐of‐pocket (OOP) health care expenses, but it is unclear how their financial burden differs by dementia stage.

**METHODS:**

We identified 2939 respondents aged ≥65 with dementia in the 2018 Health and Retirement Study, representing 9.8 million individuals on weighted analysis. We grouped respondents into four severity stages and examined their OOP expenditures, prevalence of financial risk (i.e., catastrophic or impoverishing levels of health care spending), and factors associated with financial risk.

**RESULTS:**

Individuals with severe dementia had significantly higher OOP costs, with 21% experiencing catastrophic expenditures and 12% falling below poverty thresholds due to these costs. Regression analyses indicated nursing home residence, poor subjective health, advanced age, and other factors are associated with an increased odds of financial risk.

**DISCUSSION:**

Financial risk increases in advanced dementia stages, likely reflecting more complex care needs and poorer overall health.

**Highlights:**

- Out‐of‐pocket health care costs increase with dementia severity.
- Twenty‐one percent of people with severe dementia spend at least 40% of their income on health care.
- The risk of impoverishment from health care costs increases in severe dementia.
- Care needs, poor health, and nursing home residence may contribute to financial risk.

## 1 BACKGROUND

Older adults living with dementia incur out‐of‐pocket (OOP) health care expenses that are approximately 80% higher than those without dementia.^1^ A significant proportion of these expenses is attributed to long‐term residential care, owing to patients’ loss of cognitive functioning and diminished ability to perform activities of daily living.^2^, ^3^ OOP costs for long‐term care and other services for adults with dementia are estimated to exceed $225,000 over the lifetime of the patient, and consume nearly one‐third of a household's wealth in the last 5 years of life.^1^, ^4^

Although many studies have evaluated dementia's direct and indirect costs, critical gaps remain in understanding the financial risks these costs pose for patients and their families.^1^, ^2^, ^3^, ^4^, ^5^, ^6^ Evidence from other health conditions suggests that low‐income and elderly individuals are especially vulnerable to financial hardship from OOP expenses, which can drive them into poverty.^7^, ^8^, ^9^ As catastrophic health expenditures have been linked to diminished health‐related quality of life and adverse coping strategies (e.g., forgoing basic needs, depleting assets, accruing debt), it is crucial to examine the extent to which OOP expenses for dementia care lead to catastrophic or impoverishing levels of health care spending.^10^, ^11^

Furthermore, previous analyses have often focused on aggregating costs over fixed periods of time, such as the 5–7 years preceding death^1^, ^12^ or from age 65 until death.^3^ However, care needs and expenses likely intensify as dementia becomes more severe, underscoring the importance of understanding how financial risk differs by disease stage. Such insights could enable patients and families to anticipate future expenses and inform policymakers on where financial protections are most needed. This information is also relevant for economic evaluations that assess the value of interventions aimed at delaying or reversing disease progression. As these evaluations often model progression across discrete stages of dementia, incorporating stage‐specific financial risk can provide a more comprehensive understanding of an intervention's potential to avert financial hardship resulting from high OOP spending in the later, more costly stages of dementia.

**RESEARCH IN CONTEXT**

1. **Systematic review**: The authors reviewed literature identified through biomedical databases (e.g., Medline). Although several studies have evaluated out‐of‐pocket (OOP) spending for dementia care, few have assessed the household‐level financial burden of OOP costs and how financial risk differs by dementia stage.
2. **Interpretation**: Median OOP expenditures more than double between mild and severe dementia stages. In advanced dementia, the prevalence of individuals whose OOP costs consume at least 40% of household income is higher, and an increasing proportion of individuals fall below U.S. poverty thresholds after deducting OOP expenses.
3. **Future directions**: Future studies should evaluate spillover effects of OOP expenses on families of individuals living with dementia and explore strategies to mitigate the risk of impoverishment due to healthcare expenses.

This cross‐sectional analysis examined financial risk among individuals aged 65 years and older at different stages of dementia. Specifically, we quantified OOP expenditures, estimated the prevalence of catastrophic or impoverishing health care costs, and explored the household‐ and individual‐level factors associated with financial risk.

## 2 METHODS

### 2.1 Study design and data source

We used a cross‐sectional study design to examine the prevalence and risk factors for financial hardship among older Americans with dementia. Our analysis drew upon data from the Health and Retirement Study (HRS), which is a nationally representative survey of approximately 20,000 adults aged 50 years and older (and their spouses of any age) who are living in the United States.^13^ Detailed information about health, cognition, health care costs and utilization, income, family structure, and other variables is collected on a biannual basis. At the time of analysis, data were available through the 2020 survey wave. To avoid possible confounding from the coronavirus disease 2019 (COVID‐19) pandemic's effects on healthcare utilization and OOP costs, we focused on data collected in the 2018 wave. Additionally, we conducted sensitivity analyses using data from the 2016 wave.^13^, ^14^, ^15^ The HRS is sponsored by the National Institute on Aging (grant number National Institute on Aging [NIA] U01AG009740) and is conducted by the University of Michigan.

### 2.2 Population

We restricted our analysis to individuals aged 65 years and older to focus on a Medicare‐eligible population at various stages of cognitive decline. HRS respondents were identified as having dementia if they (or a proxy respondent) reported a diagnosis of Alzheimer's disease or dementia, or were classified as having cognitive impairment or dementia using the well‐validated Langa‐Weir Classification of Cognitive Function.^16^, ^17^ The Langa‐Weir classification algorithm was developed using a sample of HRS respondents who received full, in‐person clinical evaluations as part of the Aging, Demographics, and Memory Study (ADAMS).^18^ These assessments enabled prediction of dementia status among the broader HRS survey sample by categorizing respondents into three categories: (1) *normal cognition* (i.e., respondent performs well on cognitive tests and demonstrates no significant impairment); (2) *cognitive impairment, no dementia* ([CIND]; i.e., respondent may have mild or moderate cognitive impairment that does not interfere with activities of daily living); and (3) *dementia* (i.e., respondent has severe cognitive impairment and difficulty performing activities of daily living). Following a similar approach to previous research, we required respondents meet Langa‐Weir criteria for CIND or dementia in at least two consecutive survey waves, specifically the 2018 wave and either the preceding 2016 wave, following 2020 wave, or both.^19^, ^20^, ^21^ Respondents identified with either “CIND” or “dementia” in 2018 who died before the subsequent wave were also included. Since there are known stigmas surrounding dementia, as well as disparities in the receipt of a timely diagnosis, use of the Langa‐Weir Classification helped to identify HRS respondents who might otherwise be missed by relying on self‐ or proxy‐reported diagnoses alone.^22^, ^23^

We grouped the sample into four stages of dementia severity, including (1) mild impairment, no dementia, (2) mild dementia, (3) moderate dementia, and (4) severe dementia. To assign a severity level to each respondent, we employed an empirical crosswalk that mapped the 27‐point modified Telephone Interview for Cognitive Status (TICS) to dementia stages corresponding to Mini‐Mental State Examination (MMSE) scores.^24^, ^25^ The TICS is a validated instrument for dementia screening utilized in the HRS. The crosswalk was specifically developed for use among adults 65 years and older who participate in the HRS. We categorized the mild impairment, no dementia group as having a TICS of 10–13, mild dementia as a TICS of 6–9, moderate dementia as 1–5, and severe dementia as 0 (Table S1).

In cases where individuals lacked a recorded TICS score (e.g., due to advanced dementia), we approximated their dementia severity level using the Langa‐Weir 11‐point proxy scoring system.^16^, ^17^ These criteria involve aggregating a proxy respondent's evaluation of the individual's memory, the extent of their limitations conducting instrumental activities of daily living (IADLs), and the interviewer's assessment of cognitive impairment. According to the Langa‐Weir proxy scoring system, scores of 3–5 are classified as “CIND,” which we categorized as “mild impairment, no dementia”, while scores ranging from 6 to 11 indicate “dementia.”^17^ We assumed that scores of 6–7 denoted mild dementia, 8–9 indicated moderate dementia, and scores of 10–11 represented severe dementia.

### 2.3 Outcomes

We evaluated the following measures of financial risk:

1. Out‐of‐pocket healthcare expenditures: An individual's total annual OOP health care costs summed across service categories (i.e., inpatient hospital stays, nursing home residence, in‐home medical care, ambulatory care, prescription drugs, and other healthcare expenses). All OOP cost data were adjusted to 2023 U.S. dollars using the Consumer Price Index.
2. Catastrophic health expenditures (CHE): OOP health care payments that exceed 40% of post‐subsistence annual household income.^26^, ^27^ Annual post‐subsistence household income represented total earnings and non‐job income (i.e., Social Security, pensions, welfare, interest, gifts, or other income) in the previous calendar year minus annual food costs, as reported by the Bureau of Labor Statistics.^28^ Income data were adjusted to 2023 U.S. dollars. We explored alternative catastrophic spending thresholds of 10% and 25%, which are commonly used by the World Bank, in sensitivity analyses.^11^, ^29^
3. Impoverishing health expenditures (IHE): Annual household income (as defined under CHE) that falls below the poverty line after OOP health care payments are deducted. We applied the U.S. Census Bureau's poverty thresholds, accounting for household size and family composition, and included income from institutionalized family members in these estimates.
4. Receipt of financial help from relatives (exploratory outcome): Received financial help from relatives, children, or parents in the previous 2 years totaling $500 or more. We treated this endpoint as exploratory, as we were unable to ascertain whether such assistance was associated with health care expenditures.

### 2.4 Statistical analysis

We estimated annual median (interquartile range [IQR]) OOP health expenditures stratified by dementia severity stage. As the HRS reports OOP spending over a 2‐year period, we annualized the recorded estimates. We further characterized the proportion of individuals in each dementia stage who reported OOP expenses on inpatient care (i.e., hospital stays), nursing home stays, home health services, ambulatory care (i.e., outpatient surgery and doctor visits), prescription drugs, and other medical expenses (including dental visits and visits to special facilities). For each category of care, we calculated the median dollar amount spent among those who incurred any expenditure.

To examine the prevalence of financial risk, we estimated the proportion of individuals with CHE and IHE by dementia stage. Among the subset of respondents identified with CHE, we quantified the median dollar amount by which OOP expenses exceeded the catastrophic spending threshold. Similarly, among the subset of respondents with IHE, we quantified the median dollar amount by which household income fell below the poverty threshold after deduction of OOP costs.

We fit separate regression models to assess the association between each financial risk indicator (i.e., CHE and IHE) and various household‐ and individual‐level risk factors. Although missingness was generally minimal in the dataset (i.e., < 5% across variables), we used multiple imputation with the Multivariate Imputation by Chained Equations (MICE) R package for our regression analyses. We applied a “multiple imputation then deletion” strategy in which all potential covariates and the outcome variables were used in the imputation models to generate five imputations; observations with missing outcomes were subsequently removed from the analysis to reduce noise from our estimates that would result from the imputed outcome values.^30^, ^31^

Risk factors included household‐level variables of financial wealth (i.e., the net value of all non‐housing wealth minus debt) and household size, as well as individual‐level characteristics of the person with dementia. Individual‐level factors included the need for assistance with activities of daily living (ADLs) or IADLs, self‐reported subjective health status, comorbidity burden of the respondent (i.e., total number of reported comorbidities from 0 to 9), age, sex, race, Hispanic ethnicity, marital status, educational attainment, Medicaid coverage, nursing home residence, and dementia stage (Table S2). We also developed adjusted logistic regression models to understand the association between dementia stage and financial risk, which controlled for socio‐demographic factors that could potentially confound the relationship between dementia stage and CHE or IHE, and excluded mediators that would fall along a causal pathway between the exposure and outcomes (i.e., subjective health status, assistance with ADLs or IADLs, and nursing home residence).

All analyses applied HRS sampling weights to generate nationally representative estimates. Statistical analyses were performed in R (version 4.1.0), using two‐sided statistical tests and an alpha of 0.05.

## 3 RESULTS

We identified 2939 respondents aged 65 years or older with mild impairment or dementia, which represented 9.8 million older adults on weighted analysis. Within our sample, 35% were classified as having mild impairment (no dementia), while 65% had dementia. The 65% with dementia represented approximately 11% of the U.S. population aged 65 years and older, among whom, 57% had mild dementia, 24% had moderate dementia, and 19% had severe dementia.

Table 1 presents respondents stratified by dementia stage. The mean age increased with higher levels of dementia severity, as did the proportion of female respondents. Respondents reported receiving assistance with a greater number of ADLs and IADLs at higher severity levels. A greater proportion of individuals with severe dementia resided in a nursing home and were covered by Medicaid.

**TABLE 1:** Characteristics of weighted study sample.^a^

Parameter	Mild impairment, no dementia (*n* = 2,973,990)	Mild dementia (*n* = 3,707,280)	Moderate dementia (*n* = 1,567,580)	Severe dementia (*n* = 1,224,018)	*p*‐value^b^
Age, mean (SD)	78.5 (8.1)	79.1 (8.8)	81.7 (8.7)	84.5 (8.1)	<0.001
Female, %	52.4	59.0	58.2	72.0	0.001
Race, %					0.050
White	73.5	69.8	67.7	81.0	
Black	17.0	22.1	21.8	14.9	
Other	9.5	8.1	10.6	4.1	
Hispanic, %	12.9	16.6	17.1	10.8	0.147
Currently married, %	53.6	61.4	64.8	32.6	0.008
Bachelor's degree or higher, %	11.9	8.5	10.9	17.7	0.036
Sum of ADLs where Respondent receives help, mean (SD)	0.4 (1.1)	0.5 (1.3)	1.2 (1.9)	3.8 (2.1)	<0.001
Sum of IADLs where Respondent receives help, mean (SD)	0.6 (1.1)	0.8 (1.2)	2.0 (1.8)	4.3 (1.0)	<0.001
Subjective Health, %					<0.001
Excellent	5.1	3.3	3.4	0.9	
Very good	21.2	20.9	17.2	7.7	
Good	31.7	28.2	24.2	18.7	
Fair	27.8	34.6	36.1	27.6	
Poor	14.3	12.9	19.2	45.1	
Sum of comorbidities, mean (SD)	1.4 (1.0)	1.6 (0.9)	1.4 (0.9)	1.3 (0.9)	0.010
Nursing home resident, %	3.2	4.2	11.2	42.5	<0.001
Medicaid coverage, %	16.4	21.2	27.4	37.6	<0.001
Medicaid coverage among nursing home residents^c^, %	26.2	33.2	41.8	68.9	0.020
Residents in household, mean (SD)	2.1 (1.2)	2.1 (1.3)	2.1 (1.3)	1.9 (1.1)	0.130
Household income in previous year, median [IQR]	32,820 [16,097, 63,720]	25, 361 [15,253, 48,050]	24,374 [14,918, 43,278]	29,710 [17,884, 55,399]	0.001
Household financial wealth, median [IQR]	1,149 [0, 63,038]	44 [0, 24,170]	186 [0, 24,863]	944 [0, 88,987]	0.222

Abbreviations: ADL, activities of daily living; ANOVA, analysis of variance; IADL, instrumental activities of daily living; IQR, interquartile range; SD, standard deviation.

^a^ Unweighted sample size of each severity level: mild impairment, no dementia (*n* = 1031); mild dementia (*n* = 1237); moderate dementia (*n* = 429); and severe dementia (*n* = 242).

^b^ A chi‐squared test was used to test statistical differences between frequencies, an ANOVA was used to test for differences between means, and a Kruskal‐Wallis test was used to test for differences between medians.

^c^ Estimated among the subset of individuals residing in a nursing home; the unweighted sample size of nursing home residents by dementia severity level was mild impairment, no dementia (*n* = 16); mild dementia (*n* = 30); moderate dementia (*n* = 29); and severe dementia (*n* = 72).

### 3.1 Out‐of‐pocket expenditures by dementia stage

Median total OOP spending among respondents with severe dementia was more than double that of respondents with mild impairment, no dementia (Table 2). Compared to individuals with less severe stages of dementia, a significantly greater proportion of patients with severe dementia reported recent OOP expenses on nursing home stays. While residence in a nursing home was considerably higher among the severe versus moderate dementia group (43% vs. 11%), median OOP spending on nursing home stays was lower ($4118 vs. $40,165).

**TABLE 2:** OOP expenditures in the previous year.^a^

Parameter	Mild impairment, no dementia (*n* = 2,973,990)	Mild dementia (*n* = 3,707,280)	Moderate dementia (*n* = 1,567,580)	Severe dementia (*n* = 1,224,018)	*p*‐value^b^
Total health care					
Any OOP expenses, %	83.4	75.9	70.9	79.9	0.00
Median $ [IQR]	1068 [418, 2598]	1170 [410, 3456]	1108 [471, 3369]	2853 [1052, 7144]	<0.001
Inpatient					
Any OOP expenses, %	13.6	11.6	15.1	17.8	0.156
Median $ [IQR]	590 [195, 2233]	1105 [467, 2104]	437 [194, 992]	1304 [421, 2403]	0.025
Nursing home					
Any OOP expenses, %	4.9	4.3	8.6	30.9	<0.001
Median $ [IQR]	1439 [339, 16637]	3403 [540, 14,815]	40,165 [13,638, 51,806]	4118 [1561, 15,258]	<0.001
Home health services					
Any OOP expenses, %	4.3	4.7	7.6	10.5	0.026
Median $ [IQR]	322 [314, 1189]	494 [121, 1311]	891 [487, 1800]	492 [311, 1428]	0.424
Ambulatory care					
Any OOP expenses, %	39.3	34.8	30.0	30.2	0.057
Median $ [IQR]	271 [102, 797]	298 [117, 711]	259 [91, 515]	396 [182, 1493]	0.045
Prescription drugs					
Any OOP expenses, %	66.0	59.6	57.9	52.2	0.021
Median $ [IQR]	624 [342, 1488]	717 [340, 1860]	623 [271, 1605]	960 [446, 1926]	0.027
Other health care					
Any OOP expenses, %	49.5	43.5	41.2	45.9	0.191
Median $ [IQR]	311 [151, 746]	313 [124, 931]	388 [119, 888]	497 [178, 1486]	0.062

Abbreviations: IQR, interquartile range; OOP, out‐of‐pocket.

^a^ Median OOP expenses are estimated among the subset of individuals with non‐zero OOP costs.

^b^ A chi‐squared test was used to test statistical differences between frequencies and a Kruskal‐Wallis test was used to test for differences between medians.

### 3.2 Prevalence of catastrophic or impoverishing health expenditures

**FIGURE 1:**
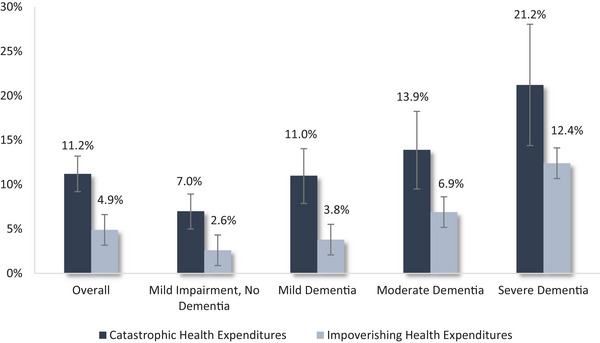
In pairwise comparisons using chi‐squared tests, differences in the prevalence of both catastrophic health expenditures and impoverishing health expenditures were statistically significant between each severity stage versus severe dementia; differences between the less severe dementia stages did not reach statistical significance. Error bars represent 95% confidence intervals.

The prevalence of catastrophic and impoverishing health expenditures increased with each dementia severity level, with statistical differences present in pairwise comparisons between each severity stage versus the severe dementia stage (Figure 1). We estimated that more than 20% of individuals with severe dementia spent > 40% of post‐subsistence household income on healthcare versus 7% of individuals with mild impairment, no dementia, and observed a consistent pattern across lower catastrophic thresholds (25% and 10%; Table S3). The median amount by which OOP expenses exceeded the catastrophic threshold also increased with each severity level, with a catastrophic gap of $6494 [IQR 3294, 16,140] in severe dementia versus $1918 [IQR 1224, 3670] for those with mild impairment, no dementia (Table S3).

Among individuals whose household income fell above national poverty thresholds, the prevalence of IHE increased with greater dementia severity, totaling 3%, 4%, 7%, and 12% for the mild impairment, no dementia, mild dementia, moderate dementia, and severe dementia groups, respectively.

### 3.3 Receipt of informal financial assistance

Table S3 reports our exploratory analysis of whether receipt of financial help from relatives increased by severity level. The proportion of individuals with severe dementia who reported receiving financial help was nearly double that of individuals in other groups (19% vs. 10% in each of the other severity levels).

### 3.4 Risk factors for catastrophic or impoverishing health expenditures

In univariable logistic regression, individuals with severe dementia had an 8.4 times greater odds of experiencing CHE (95% confidence interval [CI]: 5.2–13.5) and 10.3 times greater odds of experiencing IHE (95% CI 6.4–16.6) than individuals with mild impairment, no dementia (Table 3). In addition to dementia severity, univariable logistic regression analyses suggested that advanced age, female sex, greater need for assistance with ADLs and IADLs, poorer subjective health status, greater number of comorbidities, nursing home residence, and Medicaid coverage were all significantly associated with an increased odds of CHE and IHE. Alternatively, being currently married, having a college education, living with a greater number of household residents, and having greater financial wealth were all associated with reduced risk of CHE and IHE.

**TABLE 3:** Risk factors for catastrophic or impoverishing health expenditures.

	Catastrophic health expenditures (40% Threshold)		Impoverishing health expenditures	
Parameter	Crude OR (95% CI)	Adjusted OR (95% CI)	Crude OR (95% CI)	Adjusted OR (95% CI)
Dementia stage (Ref. mild impairment, no dementia)				
Mild dementia	1.73 (1.15–2.60)	1.46 (0.96–2.20)	1.73 (1.13–2.67)	1.44 (0.91–2.29)
Moderate dementia	2.27 (1.42–3.62)	1.58 (0.99–2.51)	2.49 (1.30–4.77)	1.72 (0.91–3.24)
Severe dementia	8.39 (5.21–13.51)	4.06 (2.44–6.76)	10.32 (6.42–16.58)	4.06 (2.43–6.77)
Age, years	1.07 (1.06–1.09)	1.05 (1.03–1.06)	1.05 (1.08–1.13)	1.07 (1.04–1.09)
Female	2.29 (1.82–2.88)	1.56 (1.18–2.04)	1.81 (1.33–2.45)	1.06 (0.74–1.53)
Race (Ref. White)				
Black	1.59 (1.05–2.40)	1.22 (0.81–1.82)	0.82 (0.50–1.35)	0.65 (0.38–1.13)
Other	1.98 (1.24–3.18)	1.76 (1.01–3.06)	0.28 (0.11–0.72)	0.38 (0.14–1.05)
Hispanic	1.95 (1.29–2.93)	1.71 (0.97–3.00)	0.69 (0.35–1.39)	0.88 (0.49–1.58)
Currently married	0.22 (0.17–0.30)	0.37 (0.28–0.50)	0.19 (0.12–0.31)	0.48 (0.27–0.87)
Bachelor's degree or higher	0.63 (0.45–0.87)	1.16 (0.84–1.61)	0.58 (0.39–0.85)	0.90 (0.60–1.36)
Number of ADLs where respondent receives help	1.55 (1.46–1.65)	–	1.57 (1.46–1.69)	–
Sum of IADLs where respondent receives help	1.67 (1.58–1.78)	–	1.73 (1.60–1.86)	–
Subjective health (Ref. Excellent)		–		–
Very good	2.73 (1.09–6.89)		0.78 (0.28–2.16)	
Good	3.99 (1.72–9.27)		1.94 (0.71–5.33)	
Fair	10.77 (4.86–23.84)		3.64 (1.32–10.00)	
Poor	14.94 (6.23–35.87)		5.28 (1.93–14.43)	
Sum of comorbidities	1.31 (1.16–1.49)	1.19 (1.04–1.37)	1.29 (1.09–1.52)	1.19 (1.00–1.42)
Nursing home resident	10.70 (7.44–15.40)	–	11.71 (7.71–17.79)	–
Medicaid coverage	2.08 (1.43–3.03)	0.91 (0.63–1.34)	2.03 (1.28–3.22)	1.10 (0.67–1.79)
Residents in household	0.72 (0.57–0.91)	0.93 (0.83–1.05)	0.31 (0.20–0.47)	0.58 (0.42–0.80)
Financial wealth (per $50,000)	0.94 (0.89–1.00)	0.96 (0.92–1.00)	0.96 (0.94–0.99)	0.96 (0.94–0.99)

Abbreviations: ADL, activities of daily living; CI, confidence interval; IADL, instrumental activities of daily living; OR, odds ratio.

– indicates variable was excluded from adjusted analyses as a mediator between relationship between dementia stage and financial risk outcomes.

In adjusted analyses, severe dementia was associated with a fourfold greater odds of CHE and IHE compared to mild impairment, no dementia (Table 3), although less advanced dementia stages did not statistically differ from the mild impairment, no dementia group.

### 3.5 Sensitivity analyses

Tables S4–S7 present the results of our sensitivity analyses using the 2016 survey wave. Findings were generally consistent across waves, showing higher total OOP expenses and an increased prevalence of CHE and IHE in severe dementia. However, nursing home expenditures differed between waves; while data from 2018 suggested individuals with moderate dementia had the highest OOP spending on nursing home stays, the 2016 data indicated that these costs were greatest in the severe stage.

## 4 DISCUSSION

This study examined the prevalence and risk factors for financial risk among individuals with dementia, revealing that OOP healthcare expenses, as well as the prevalence of CHE and IHE, were significantly higher among older adults with severe dementia compared to those with less severe stages of dementia or cognitive impairment. In multivariable‐adjusted regression analyses, severe dementia was associated with an increased odds of CHE and IHE, suggesting that financial vulnerability from OOP costs worsens as the disease progresses.

Among the general sample of 2018 HRS respondents aged 65 years and older, 4.4% experienced CHE, and 2.0% experienced IHE. In contrast, our estimates suggest a substantially higher prevalence of CHE (11.2% overall, ranging from 7% to 21% across dementia stages) and IHE (4.9% overall, 3%–12% across dementia stages) among dementia households. This heightened financial risk likely reflects the increased caregiving and health care demands associated with dementia. In univariable regression analysis, indicators of greater care needs, such as nursing home residence and requiring assistance with ADLs and IADLs, were strongly linked to CHE and IHE, particularly in more advanced disease stages.

While Medicaid can help alleviate financial hardship from OOP costs by providing more generous coverage for long‐term care, eligibility often requires individuals to deplete their assets, which may have unintended long‐term financial consequences for families. Our study found that individuals with moderate dementia incurred higher OOP nursing home costs than those with severe dementia, despite greater care needs and nursing home use in severe dementia. This finding may indicate that individuals with moderate dementia were in the process of “spending down” their income on health care expenses to meet Medicaid eligibility requirements. However, further investigation is warranted, as this pattern was not observed in the sensitivity analysis of the 2016 survey wave.^32^ Private long‐term care insurance, which is also meant to mitigate the financial burdens of nursing home care and other services, has had low uptake in the United States, perhaps due to the high perceived cost of premiums.^33^

Given the challenges in accessing financial protection mechanisms, research on the economic burden of dementia must go beyond absolute cost estimates to examine how healthcare expenses affect patients and their families. Measures such as CHE and IHE help contextualize OOP spending relative to financial capacity, providing insights into the strain health care costs place on household resources as well as their potential to drive families into poverty. Quantifying these indicators across disease stages can inform the design of more targeted interventions and refinements to social safety nets, and strengthen economic evaluations of new therapies by capturing their potential to prevent or delay progression to more financially burdensome stages. Greater awareness of how financial risks vary by dementia severity may also help patients and families plan for future care needs and associated expenses.

Although our cross‐sectional study identified stage‐specific differences in financial risk, future research should examine how financial vulnerability evolves over time as dementia progresses. Longitudinal analysis, as well as qualitative studies that explore how families experience, adapt to, and manage financial strain throughout the disease course, could enrich the insights gleaned from this study. In particular, understanding the impact of varying levels of catastrophic spending on the physical, psychological, and financial well‐being of patients and their families remains crucial, including examining the extent to which such spending necessitates tradeoffs in basic needs or compels households to adopt other coping strategies.

Future research should also examine appropriate catastrophic health expenditure thresholds for a U.S. Medicare population, with particular attention to whether distinct thresholds are needed for institutionalized versus community‐dwelling populations. As nursing home costs replace certain living expenses, such as meals and housing, differing thresholds may be warranted. However, it remains essential to account for the broader family‐level implications of OOP expenses, with appropriate adjustments for Medicaid coverage.

### 4.1 Limitations

There were some limitations to note with this study. First, we relied on self‐reported diagnoses and algorithmic classification of respondents’ dementia status using the Langa‐Weir criteria. Due to disparities in dementia diagnoses, algorithmic classification helps include individuals potentially missed by self‐reports, though this carries a risk of false positives.^22^, ^23^ To mitigate this risk, we required individuals meet Langa‐Weir criteria across at least two survey waves.

Additionally, without detailed clinical evaluations, we could not definitively classify respondents into dementia severity levels or ascertain their specific type of dementia. We relied on an empirical crosswalk between two cognitive impairment instruments, which may have resulted in some misclassification, though respondents’ characteristics aligned with expected patterns (i.e., increased age, greater difficulty with activities of daily living, poorer health status, and higher rates of nursing home residence in advanced stages). To further assess the validity of our severity classifications, we compared the distribution of individuals across severity levels in our sample to that of other studies (see Table S8). While differences in study populations and time periods exist, our estimates approximated those from a Framingham Heart Study publication in which investigators had access to health records and used an expert review panel to classify dementia stage.^34^

Since HRS data are biennial, we annualized OOP expenditures, which may have misclassified financial risk for those with concentrated expenses in 1 year, potentially underestimating risk prevalence.

Lastly, our IHE analysis excluded individuals already below the poverty threshold who may have been further impoverished by their care expenses.

## 5 CONCLUSION

Our study highlights the significant financial burden faced by older adults with dementia, particularly in the advanced stages of the disease. The interplay between intensified caregiving needs, poorer health, and socioeconomic factors such as Medicaid coverage shapes these financial risks. These findings emphasize the need for targeted policy interventions to mitigate the economic challenges faced by this vulnerable population and to better prepare families for the financial demands of dementia care.

## CONFLICT OF INTEREST STATEMENT

Patricia Synnott, Pei‐Jung Lin, and Yingying Zhu are employed at the Center for the Evaluation of Value and Risk in Health at the Institute for Clinical Research and Health Policy Studies at Tufts Medical Center, which receives funding from government, private foundation, and pharmaceutical industry sources in support of initiatives outside the submitted work; these sources include the National Institute on Aging, Biogen, Eisai, Eli Lilly, Genentech, GSK, Incyte, and Argenx. Pei‐Jung Lin reported personal fees from Biogen and Halozyme outside the submitted work. Angie Mae Rodday has no disclosures related to the content of the manuscript. Author disclosures are available in the supporting information.

## Supporting information



Supporting Information

Supporting Information
