# Supplementary material for: Characterizing financial risk from out‐of‐pocket expenditures across dementia stages
Source: Alzheimers Dement. 2025 Sep 15;21(9):e70666. doi: 10.1002/alz.70666 (PMC12434702; doi:10.1002/alz.70666)
Supplement: Supplementary file 2 — Supporting Information [file ALZ-21-e70666-s002.docx]

**Characterizing Financial Risk from Out-of-Pocket Expenditures across Dementia Stages**

***Appendix***

[**Section 1. Supplemental Tables** 2](#_Toc206054728)

[Supplemental Table 1. Categorization of Dementia Severity Level 2](#_Toc206054729)

[Supplemental Table 2. Description of Key Covariates 3](#_Toc206054730)

[Supplemental Table 3. Prevalence of Financial Risk 4](#_Toc206054731)

[**Section 2. Sensitivity Analysis from the 2016 Health and Retirement Study** 5](#_Toc206054732)

[Supplemental Table 4. Characteristics of Weighted Study Samples in 2016 and 2018 5](#_Toc206054733)

[Supplemental Table 5. Out of Pocket Expenditures in the 2016 and 2018 Survey Waves* 7](#_Toc206054734)

[Supplemental Table 6. Prevalence of Financial Risk in the 2016 and 2018 Survey Waves 9](#_Toc206054735)

[Supplemental Table 7. Distribution of Dementia Stages in the 2016 and 2018 Survey Waves 10](#_Toc206054736)

[**Section 3. Distribution of Dementia Severity Stages** 11](#_Toc206054737)

[Supplemental Table 8. Comparison of Severity Distribution with Other Studies 11](#_Toc206054738)

[**References** 12](#_Toc206054739)

**Section 1. Supplemental Tables**

**Supplemental Table 1. Categorization of Dementia Severity Level**

|  | **Cognitive Impairment level** | | | **Dementia Status** |
| --- | --- | --- | --- | --- |
|  | **MMSE** | **TICS-27** | **Langa-Weir Proxy Score** |  |
| **Severe dementia** | 0-10 | 0 | 10-11 | Self- or proxy-reported diagnosis of dementia, or Langa-Weir classification of CIND or dementia |
| **Moderate dementia** | 11-20 | 1-5 | 8-9 |  |
| **Mild dementia** | 21-24 | 6-9 | 6-7 |  |
| **Mild impairment, no dementia** | 25-27 | 10-13 | 3-5 |  |
| CIND=Cognitive impairment, no dementia; MMSE=Mini-Mental State Examination; TICS=Telephone Interview for Cognitive Status | | | | |

**Supplemental Table 2. Description of Key Covariates**

| **Variable** | **Description** |
| --- | --- |
| Household wealth | Net value of all non-housing wealth (real estate, vehicles, businesses, retirement accounts, stocks/mutual funds/investment trusts, checking/savings/money market accounts, CD/government savings bonds/T-bills, bonds and bond funds, other savings) minus debt |
| Household income | Total household earnings and non-job income (i.e., Social Security, pensions, welfare, interest, gifts, or other income) in the previous calendar year |
| Household size | Number of people living in household |
| Comorbidity burden | Total number (0-9) of doctor-diagnosed health problems at time of interview: high blood pressure, diabetes, cancer, lung disease, heart problems, stroke, psychological problems, arthritis, sleep disorder |
| Age | Age (years) at time of interview |
| Sex | Male, female |
| Race | White/Caucasian; Black/African American; Other |
| Ethnicity | Hispanic; Not Hispanic |
| Marital status | Currently married; Not married (partnered, separated, divorced, widowed, or never married) |
| Educational attainment | Bachelor’s degree or higher (yes/no) |
| Insurance status | Respondent is covered by Medicaid (yes/no) |
| Residence | Lives in nursing home at time of interview (yes/no) |
| Dementia stage | Cognitive impairment, no dementia; mild dementia; moderate dementia; severe dementia |

**Supplemental Table 3. Prevalence of Financial Risk**

|  | **Overall**  (n=9,761,817) | **Mild Impairment, No Dementia**  (n=2,973,990) | **Mild Dementia**  (n=3,707,280) | **Moderate Dementia**  (n=1,567,580) | **Severe Dementia**  (n=1,224,018) | **p*** |
| --- | --- | --- | --- | --- | --- | --- |
| **Catastrophic health expenditures** |  |  |  |  |  |  |
| **40% threshold, %** | 11.2 | 7.0 | 11.0 | 13.9 | 21.2 | <0.001 |
| **Catastrophic gap, median [IQR]** | 3,592 [1,445, 10,697] | 1,918 [1,224, 3,670] | 3,258 [1,068, 9,015] | 3,899 [1,355, 30,203] | 6,494 [3,294, 16,140] | 0.001 |
| **25% threshold, %** | 14.7 | 9.8 | 14.8 | 17.1 | 25.1 | <0.001 |
| **Catastrophic gap, median $ [IQR]** | 3,400 [1,392, 9,038] | 1,874 [1,268, 4,214] | 3,043 [1,196, 7,207] | 4,707 [1,266, 29,222] | 5,324 [2,965, 13,776] | <0.001 |
| **10% threshold, %** | 26.5 | 20.8 | 26.4 | 29.4 | 39.5 | <0.001 |
| **Catastrophic gap, median $ [IQR]** | 1,964 [574, 5,755] | 1,197 [231, 4,093] | 2,221 [690, 4,735] | 1,993 [599, 6,931] | 2,812 [958 , 11,649] | <0.001 |
| **Impoverishing health expenditures, %** | 4.9 | 2.6 | 3.8 | 6.9 | 12.4 | <0.001 |
| **Impoverishment gap, median $ [IQR]** | 5,469 [1,611 22,288] | 2,116 [822, 13,370] | 2,375 [425, 6,454] | 18,106 [4,382, 40,307] | 9,703 [1,570, 45,518] | 0.017 |
| **Receipt of financial help from relatives,^†^ %** | 11.5 | 10.3 | 9.8 | 9.8 | 18.9 | 0.012 |

*A chi-square test was used to test statistical differences between frequencies and a Kruskal-Wallis test was used to test for differences between medians

**^†^**Reported receiving financial help from relatives, children, or parents in last two years totaling $500 or more

**Section 2. Sensitivity Analysis from the 2016 Health and Retirement Study**

**Supplemental Table 4. Characteristics of Weighted Study Samples in 2016 and 2018**

|  | **2016**  (n=10,336,375) | **2018**  (n=9,761,817) |
| --- | --- | --- |
| **Age, mean (SD)** | 80.2 (8.8) | 79.9 (8.7) |
| **Female, %** | 58.6 | 58.5 |
| **Race, %** |  |  |
| **White** | 76.3 | 72.6 |
| **Black** | 16.8 | 19.1 |
| **Other** | 7.0 | 8.3 |
| **Hispanic, %** | 13.2 | 14.5 |
| **Currently married, %** | 40.0 | 40.0 |
| **Bachelor’s degree or higher, %** | 12.7 | 12.1 |
| **Sum of ADLs where Respondent receives help, mean (SD)** | 1.4 (2.2) | 1.5 (2.0) |
| **Sum of IADLs where Respondent receives help, mean (SD)** | 1.4 (1.8) | 1.4 (1.8) |
| **Subjective Health, %** |  |  |
| **Excellent** | 4.7 | .3.5 |
| **Very good** | 16.7 | 18.5 |
| **Good** | 28.1 | 27.7 |
| **Fair** | 31.9 | 31.9 |
| **Poor** | 18.6 | 18.5 |
| **Sum of Comorbidities, mean (SD)** | 1.5 (0.9) | 1.5 (0.9) |
| **Nursing home resident, %** | 14.2 | 10.0 |
| **Medicaid Coverage, %** | 21.3 | 22.7 |
| **Nursing home resident with Medicaid, %** | 35.1 | 55.0 |
| **Residents in household, mean (SD)** | 2.0 (1.2) | 2.0 (1.2) |
| **Household income in previous year, median [IQR]*** | 23,340 [14,112, 42,807] | 23,501 [13,246, 43,624] |
| **Household financial wealth, median [IQR]*** | 1,200 [0, 50,000] | 500 [0, 35,000] |

ADL=Activity of Daily Living; IADL=Instrumental Activity of Daily Living; IQR=interquartile range; SD=standard deviation

*Values reflect nominal U.S. dollars

**Supplemental Table 5. Out of Pocket Expenditures in the 2016 and 2018 Survey Waves***

| **Median $ [IQR]** | **Mild Impairment, No Dementia** | **Mild Dementia** | **Moderate Dementia** | **Severe Dementia** | **p**^†^ |
| --- | --- | --- | --- | --- | --- |
| **Total healthcare** |  |  |  |  |  |
| 2016 | 1,070 [383, 2,558] | 1,261 [459, 3,809] | 1,525 [503, 4,505] | 5,295 [1,055, 18,89] | <0.001 |
| 2018 | 1,068 [418, 2,598] | 1,170 [410, 3,456] | 1,108 [471, 3,3695] | 2,853 [1,052, 7,144] | <0.001 |
| **Inpatient** |  |  |  |  |  |
| 2016 | 527 [198, 1,213] | 721 [351, 1,684] | 657 [189, 1,646] | 873 [424, 2,197] | 0.184 |
| 2018 | 590 [195, 2,233] | 1,105 [467, 2,104] | 437 [194, 992] | 1,304 [421, 2,403] | 0.025 |
| **Nursing home** |  |  |  |  |  |
| 2016 | 1,287 [464, 5,057] | 5,468 [1,067, 17,433] | 3,365 [925, 15,922] | 11,520 [4,751, 30,827] | 0.008 |
| 2018 | 1,439 [339, 16,637] | 3,403 [540, 14,815] | 40,165 [13,638, 51,806] | 4,118 [1,561, 15,258] | <0.001 |
| **Home health services** |  |  |  |  |  |
| 2016 | 1,211 [233, 2,937] | 450 [258, 2,681] | 543 [95, 3,507] | 1,003 [564, 3,229] | 0.579 |
| 2018 | 322 [314, 1,189] | 494 [121, 1,311] | 891 [487, 1,800] | 492 [311, 1,428] | 0.424 |
| **Ambulatory care** |  |  |  |  |  |
| 2016 | 255 [89, 790] | 333 [109, 1,305] | 333 [105, 1,269] | 277 [102, 912] | 0.197 |
| 2018 | 271 [102, 797] | 298 [117, 711] | 259 [91, 515] | 396 [182, 1,493] | 0.045 |
| **Prescription drugs** |  |  |  |  |  |
| 2016 | 582 [264, 1,360] | 681 [323, 1,656] | 735 [335, 1,836] | 984 [355, 2,040] | 0.013 |
| 2018 | 624 [342, 1,488] | 717 [340, 1,860] | 623 [271, 1,605] | 960 [446, 1,926] | 0.027 |
| **Other** |  |  |  |  |  |
| 2016 | 276 [123, 906] | 306 [116, 909] | 398 [143, 940] | 461 [152, 1,308] | 0.089 |
| 2018 | 311 [151, 746] | 313 [121, 931] | 388 [119, 888] | 497 [178, 1,486] | 0.062 |

IQR=interquartile range; OOP=out-of-pocket

*****Median OOP expenses are estimated among the subset of individuals with non-zero OOP costs

^†^A Kruskal-Wallis test was used to test for differences between medians

**Supplemental Table 6. Prevalence of Financial Risk in the 2016 and 2018 Survey Waves**

|  | **Overall** | **Mild Impairment, No Dementia** | **Mild Dementia** | **Moderate Dementia** | **Severe Dementia** | **p*** |
| --- | --- | --- | --- | --- | --- | --- |
| **Catastrophic health expenditures, %** | | | | | | |
| **2016** | 11.7 | 7.3 | 10.2 | 14.5 | 30.0 | <0.001 |
| **2018** | 11.2 | 7.0 | 11.0 | 13.9 | 21.2 | <0.001 |
| **Impoverishing health expenditures, %** | | | | | | |
| **2016** | 5.6 | 2.7 | 3.8 | 6.2 | 20.0 | <0.01 |
| **2018** | 4.9 | 2.6 | 3.8 | 6.9 | 12.4 | <0.001 |

*A chi-square test was used to test statistical differences between frequencies

**Supplemental Table 7. Distribution of Dementia Stages in the 2016 and 2018 Survey Waves**

|  | **Mild Dementia** | **Moderate Dementia** | **Severe Dementia** |
| --- | --- | --- | --- |
| **2016** | 53% | 26% | 21% |
| **2018** | 57% | 24% | 19% |

**Section 3. Distribution of Dementia Severity Stages**

**Supplemental Table 8. Comparison of Severity Distribution with Other Studies**

|  | **Current Study** | **Tahami Monfared, J Prev Alzheimers Dis, 2024^1^** | | **Yuan, J Alzheimers Dis, 2021^2^** |
| --- | --- | --- | --- | --- |
| **Severity** **Distribution** |  | **Self-Reported AD Sample** | **TICS score alone Sample** |  |
| Mild Dementia | 57% | 27% | 53% | 50% |
| Moderate Dementia | 24% | 42% | 40% | 30% |
| Severe Dementia | 19% | 31% | 7% | 19% |
| **Study Elements** |  |  |  |  |
| Data Source | Health and Retirement Study (2018) | Health and Retirement Study (2014, 2016, 2018* and 2020)  *2018 estimates recorded in rows above | | Framingham Heart Study (2004-2009) |
| Age (years) | 65+ | >=50 | | 50-94 |
| Approach to Dementia Classification | Self- or proxy-reported diagnosis of Alzheimer’s disease or other type of dementia, and/or Langa Weir algorithm in 2 subsequent survey waves | Self-report of Alzheimer’s disease | TICS score ≤17 (available for 46% of respondents) | AD diagnosis based on examination of health records |
| Severity Staging | TICS score mapped to MMSE or Langa-Weir Proxy Scores | TICS score (available for 30% of respondents) mapped to MMSE | TICS irrespective of Alzheimer’s disease diagnosis (available for 46% of respondents) mapped to MMSE | Clinical judgment by review panel |
| AD: Alzheimer’s disease; MMSE: Mini-Mental State Examination; TICS: Telephone Interview for Cognitive Status | | | | |

**References**

1. Tahami Monfared AA, Hummel N, Chandak A, Khachatryan A, Zhang R, Zhang Q. Prevalence Estimation of Dementia/Alzheimer's Disease Using Health and Retirement Study Database in the United States. *J Prev Alzheimers Dis.* 2024;11(5):1183-1188.

2. Yuan J, Maserejian N, Liu Y, et al. Severity Distribution of Alzheimer's Disease Dementia and Mild Cognitive Impairment in the Framingham Heart Study. *J Alzheimers Dis.* 2021;79(2):807-817.
